# Biofilm-Forming Capability of Highly Virulent, Multidrug-Resistant *Candida auris*

**DOI:** 10.3201/eid2302.161320

**Published:** 2017-02

**Authors:** Leighann Sherry, Gordon Ramage, Ryan Kean, Andrew Borman, Elizabeth M. Johnson, Malcolm D. Richardson, Riina Rautemaa-Richardson

**Affiliations:** University of Glasgow, Glasgow, Scotland, UK (L. Sherry, G. Ramage, R. Kean);; Public Health England, Bristol, UK (A. Borman, E.M. Johnson);; The University of Manchester, Manchester, UK (M.D. Richardson, R. Rautemaa-Richardson);; University Hospital of South Manchester, Manchester (M.D. Richardson, R. Rautemaa-Richardson)

**Keywords:** Candida auris, yeast, fungi, virulent, antifungal drug­ resistant, multidrug resistant, biofilm, candidemia, killing assay, aggregative phenotypes, nonaggregative phenotypes, healthcare–associated infections, chlorhexidine

## Abstract

The emerging multidrug-resistant yeast pathogen *Candida auris* has attracted considerable attention as a source of healthcare–associated infections. We report that this highly virulent yeast has the capacity to form antifungal resistant biofilms sensitive to the disinfectant chlorhexidine in vitro.

The yeast pathogen *Candida auris* was first detected in 2009 from an ear canal infection in Japan (*1*). This species initially attracted attention because of its reduced susceptibility to azoles and amphotericin B, combined with the lack of reliable culture-based methods for its identification (*2*). More recently, *C. auris* has been associated globally with life-threatening invasive diseases, such as bloodstream and wound infections. *C. auris* has also caused hospital outbreaks across Asia and South America, as highlighted in a 2016 clinical alert (*3*). In addition, in a UK intensive care unit, candidemia developed in 20% of patients colonized with *C. auris* (*4*). Although the mode of transmission within hospitals is unknown, *C. auris* may substantially contaminate rooms of colonized or infected patients (*5*). Phospholipase and proteinase activity have been identified as virulence factors (*6*); however, because previously used assessment techniques were rudimentary, this pathogen’s ability to form biofilm remains under question (*7*). The draft genome identifying various proteins involved in biofilm formation (*8*), coupled with recent descriptions of aggregative and nonaggregative phenotypes, the latter of which are more virulent in vivo (*9*), indicate the possibility of heterogeneous *C. auris* biofilm formation, as described for *C. albicans* (*10*). We sought to examine these aggregative and nonaggregative *C. auris* phenotypes in the context of biofilm-forming capacity, investigate their susceptibility to a panel of antifungal agents and the skin disinfectant chlorhexidine, and investigate their virulence in vivo*.*

## The Study

**Figure 1:**
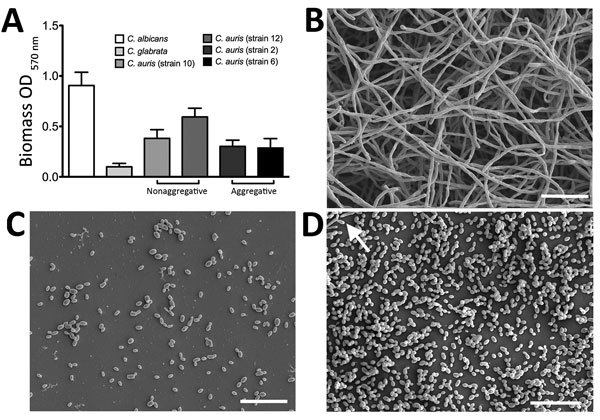
Biofilm formation on *Candida auris*, *C. albicans*, and *C. glabrata* yeast strains. A) Biomass quantities were determined spectrophotometrically for 4 strains of *C. auris* and 1 each of *C. albicans* and *C. glabrata*. Isolates were standardized to 10^6^ cells/mL in RPMI-1640 and grown in flat-bottomed 96-well microtiter plates for 24 h at 37°C. Biofilms were then washed, stained with crystal violet solution, and quantified. Data represent the mean ± SD of experiments performed on 3 separate occasions, using 8 replicates for each strain. Results show that *C. auris* can form heterogeneous intermediate biofilms. B–D) *C. albicans* (B), *C. glabrata* (C) and *C. auris* (D) were also grown on Thermanox coverslips (Thermo Fisher Scientific, Paisley, UK) for 24 h at 37°C. Biofilms were then processed and viewed on a JEOL (Tokyo, Japan) JSM-6400 scanning electron microscope; images were assembled using Photoshop software (http://www.photoshop.com/products). Arrow indicates pseudohyphae in *C. auris* biofilm (D). Scale bars represent 20 μm (original magnification ×1,000). OD, optical density.

Throughout this study, we used *C. albicans* SC5314 and *Candida glabrata* WT2001 as comparators for *C. auris* nonaggregative strains NCPF 8971 (strain 10) and NCPF 8973 (strain 12) and aggregative strains NCPF 8977 (strain 2) and NCPF 8978 (strain 6), as previously described (*9*). Strains were propagated in YPD broth (Sigma-Aldrich, Dorset, UK), incubated overnight at 30°C, and adjusted to 10^6^ cells/mL in RPMI 1640 medium (*11*). On 3 separate occasions, 8 biofilms of each *Candida* species were grown in flat-bottomed, 96-well polystyrene microtiter plates and incubated for 24 h at 37°C, after which biomass was assessed by crystal violet assay (*12*). *C. albicans* displayed the greatest biofilm mass (Figure 1, panel A), consistent with previous findings (*10*). Compared with *C. albicans*, all *C. auris* strains formed significantly reduced biofilms (p<0.0001): biomass for nonaggregative *C. auris* strains 10 and 12 was 2.4 and 1.5 times less, respectively, than those for *C. albicans*, and biomass for aggregative *C. auris* strains 2 and 6 was 3.0 and 3.1 times less, respectively. However, these strains formed significantly greater biofilms (p<0.0001) than those formed by *C. glabrata* (3.8, 6.0, 3.0, and 2.9 times more for strains 10, 12, 2, and 6, respectively). We confirmed these findings for each species by scanning electron microscopy after growing the strains on Thermanox Coverslips (Thermo Fisher Scientific, Paisley, UK) for 24 h, as previously described (*12*). *C. albicans* biofilms were typically densely packed with hyphae (Figure 1, panel B), whereas *C. glabrata* formed a sparse biofilm consisting of yeast cells only, without extracellular matrix (Figure 1, panel C). *C. auris* strain 10 biofilm formation was intermediate to the *C. albicans* and *C. glabrata* phenotypes, showing predominately budding yeast and occasional pseudohyphae (Figure 1, panel D). In agreement with previous findings (*9*), all tested *C. auris* strains displayed the same phenotype.

To determine MICs for planktonic and sessile cells of the *C. auris* strains, we performed antifungal susceptibility testing using standardized Clinical Laboratory Standards Institute M27-A3 broth microdilution (visual inspection) and standardized candidal biofilm testing (metabolic viability) with fluconazole, voriconazole, caspofungin, micafungin, liposomal amphotericin B, amphotericin B, and chlorhexidine (*13*,*14*). Antifungal agents were tested in serial 2-fold dilutions (0.06–32.0 mg/L) for planktonic and sessile cells. Fluconazole was ineffective (MICs of >32 mg/L) against planktonic and sessile communities, whereas voriconazole displayed minimal activity against planktonic cells (Table 1, 2). Although liposomal amphotericin B was active against planktonic *C. auris* at 0.25–1.0 mg/L, up to 16 mg/L was required to reduce biofilm metabolic viability by 90%. Amphotericin B was more effective, requiring 4 mg/L to kill biofilms. Micafungin was the most active echinocandin, requiring <0.5 mg/L to inhibit planktonic cells, compared with 2–32 mg/L for caspofungin. However, these 2 antifungal agents were ineffective against biofilms, requiring >32 mg/L to inhibit sessile cells. Of note, chlorhexidine exhibited the greatest activity, requiring <0.02% to effectively inhibit planktonic and sessile cells across all strains tested. All strains showed similar sensitivity profiles, with the exception of strain 10, for which voriconazole was required in higher concentrations and caspofungin in lower concentrations to effectively inhibit planktonic growth.

**Table 1:** Planktonic susceptibility profiles of 7 antifungals against *Candida auris* yeast

Drug	Planktonic MIC*			
Strain 2	Strain 6	Strain 10	Strain 12
Fluconazole	>32	>32	>32	>32
Voriconazole	8	8	32	1
Caspofungin	32	32	2	>32
Micafungin	0.5	<0.0625	<0.06	<0.0625
Liposomal amphotericin B	0.25	0.25	0.5	1
Amphotericin B	0.25	0.25	0.5	0.5
Chlorhexidine, %	<0.02	<0.02	<0.02	<0.02

*Values are mg/L except as indicated. All MIC tests were performed on 3 independent occasions, showing identical results each time.

**Table 2:** Sessile susceptibility profiles of 7 antifungals against *Candida auris* yeast

Drug	Sessile MIC*			
	Strain 2	Strain 6	Strain 10	Strain 12
Fluconazole	>32	>32	>32	>32
Voriconazole	>32	>32	>32	>32
Caspofungin	>32	>32	>32	>32
Micafungin	>32	>32	0.25	>32
Liposomal amphotericin B	2	8	16	16
Amphotericin B	2	4	2	4
Chlorhexidine, %	<0.02	<0.02	<0.02	<0.02

*Values are mg/L except as indicated. Sessile MICs are defined as a 90% inhibition of the metabolic dye XTT, 2,3-Bis(2-methoxy-4-nitro-5-sulfophenyl)-2*H*-tetrazolium-5-carboxanilide inner salt (Sigma-Aldrich, Dorset, UK) compared with the untreated control; MIC tests were performed on 3 independent occasions and showed identical results each time.

**Figure 2:**
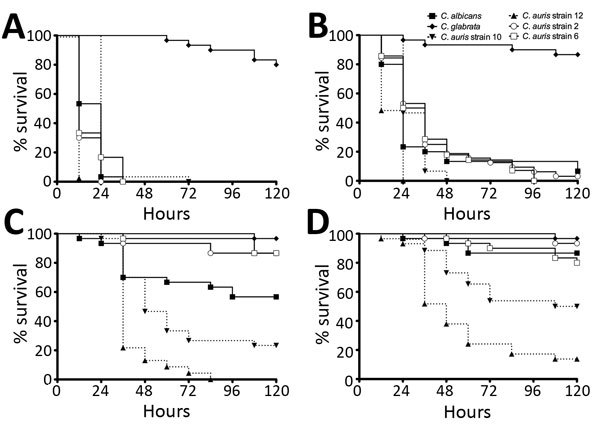
Pathogenicity of *Candida* species yeast infections in vivo. *Galleria mellonella* larvae were infected with 10^6^ (A), 5 × 10^5^ (B), 10^5^ (C), and 5 × 10^4^ (D) cells/larvae of *C. albicans*, *C. glabrata*, and 4 *C. auris* strains, and larvae survival measured every 12 h over 5 d. Ten samples of each yeast were used, and experiments were performed on 3 independent occasions. Data represents the mean percentage survival, as determined using a Kaplan-Meier plot. PBS and controls, which were pierced only, were also included and had no effect on larvae survival. Results show that *C. auris* and *C. albicans* infection exhibit similar pathogenicity.

Killing assays in *Galleria mellonella* were performed, as previously described (*12*), to assess the pathogenicity of each *Candida* species. Ten *G. mellonella* larvae (Livefoods Direct Ltd, Sheffield, UK) with bodyweights of ≈300 mg were used for each test group. Standardized inoculums of 10^6^ and 10^5^ and to 5 × 10^5^ and 5 × 10^4^ cells/larvae (Figure 2) in PBS, were injected into the hemocoel, as previously described (*9*). We assessed pathogenicity using a Kaplan-Meier plot, monitoring the percent survival over 5 days. Survival data for 5 × 10^5^ cells/larvae showed a significant difference in the killing of larvae by *C. glabrata* and the other *Candida* species (p<0.0001) (Figure 2, panel B). Although *C. albicans* and *C. auris* had similar kill kinetics in this model, infection with nonaggregative *C. auris* strain 10 achieved a 100% death rate within 48 h, compared with a rate of ≈87% with *C. albicans* (p = 0.3076). Moreover, nonaggregative *C. auris* was significantly more pathogenic than *C. albicans* when a lower inoculum of 10^5^ (p<0.05) and 5 × 10^4^ cells/larvae (p<0.01) was administered. These data, along with those of Borman et al. (*9*), suggest that the nonaggregative *C. auris* phenotype has the capacity to form biofilms with enhanced virulence capacity.

## Conclusions

Biofilm formation is a key driver of *C. albicans* pathogenicity and is associated with patient death (*10*,*15*). We show that *C. auris* can differentially adhere to polymeric surfaces, form biofilms, and resist antifungal agents that are active against its planktonic counterparts. Of particular interest, caspofungin was predominately inactive against *C. auris* biofilms; this finding was unexpected because caspofungin is normally highly effective against *Candida* biofilms. These features contribute not only to *C. auris* virulence but also to its survival in hospital environments, increasing its ability to cause outbreaks (*5*). The results of the in vivo model used in this study are in line with our clinical experience and validated by findings in other in vivo studies (*9*), affirming that *C. auris* is highly virulent or more virulent than *C. albicans*.

Although unable to form biofilms equivalent to *C. albicans*, *C. auris* has a noteworthy virulence capacity that merits further exploration, particularly given the apparent heterogeneity associated with aggregative capacity. These factors, together with the innate resistance of *C. auris* to most antifungal agents, may explain why it is an emerging pathogen. Our findings suggest it is improbable that the spread and prevalence of *C. auris* can be controlled with antifungal stewardship approaches alone. We showed that chlorhexidine is effective against *C. auris* planktonic and sessile communities. Thus, use of this disinfectant can be advocated for topical control of *C. auris* at standard concentrations used for skin and wound cleansing and disinfection (0.05%–4.0%). Infection-prevention measures targeting *C. auris* biofilms in patients, on medical devices (e.g., equipment in contact with patients), and in the hospital environment will be required.
